# Triptolide inhibits Epstein-Barr nuclear antigen 1 expression by increasing sensitivity of mitochondria apoptosis of nasopharyngeal carcinoma cells

**DOI:** 10.1186/s13046-018-0865-5

**Published:** 2018-08-15

**Authors:** Heng Zhou, Yu Liu, Chao Wang, Limei Liu, Huan Wang, Yaqian Zhang, Cong Long, Xiaoping Sun

**Affiliations:** 1 The State Key Laboratory of Virology, Department of Pathogen Biology, School of Basic Medical Sciences, Hubei Province Key Laboratory of Allergy and Immune-related Diseases, Wuhan University, 185 Eastlake Road, Wuhan, 430071 People’s Republic of China; 2 Department of Pathology, Renmin Hospital of Wuhan University, Wuhan University, Wuhan, 430060 People’s Republic of China; 3 Department of Pathogen Biology, Weifang Medical University, Weifang, 261053 People’s Republic of China; 4 Department of corneal disease, Weifang Eye Hospital, Weifang, 261041 People’s Republic of China

**Keywords:** EBNA1, Triptolide, NPC, Apoptosis, Caspase-9

## Abstract

**Background:**

Epstein-Barr virus (EBV) is widely found in nasopharyngeal carcinoma (NPC) tissue and associated with poor prognosis of patients. EBV nuclear antigen 1 (EBNA1) is expressed in all NPC tumors and plays multiple biological roles in both virus and host cells. Triptolide is a natural product extracted from *Tripterygium* and shows anti-cancer activities. The goal of this work was to illustrate the anti-cancer effect of triptolide and elucidate a novel anti-apoptotic mechanism of EBNA1 in NPC cells encountered with triptolide.

**Methods:**

In the present study, a CCK-8 assay was used to analyze the proliferation of NPC cells treated with triptolide in a dose- and time-dependent ways. Effects of triptolide on NPC cell cycle and apoptosis were investigated by flow cytometric analysis. EBNA1 expression in mRNA and protein levels was determined by quantitative real-time PCR and Western blot, respectively.

**Results:**

Our results showed that triptolide effectively inhibited proliferation of NPC cells. Triptolide arrested NPC cell cycles in S phase and induced apoptosis through a caspase-9-dependent apoptosis pathway. Low-dose of triptolide reduced the half-life of EBNA1 and significantly decreased EBNA1 expression by promoting the process of proteasome-ubiquitin pathway. Over-expression of EBNA1, which was independent from EBV genome, effectively attenuated the apoptosis induced by triptolide. In addition, triptolide significantly inhibited proliferations of tumors induced by EBV-positive cells in vivo. Furthermore, EBNA1 were expressed in all NPC biopsies of Chinese patients.

**Conclusions:**

In summary, our study provides the evidence that triptolide induces EBNA1 degradation and stimulates NPC apoptosis through mitochondria apoptotic pathway. In addition, EBNA1 assists NPC cells to resist triptolide-induced apoptosis through inhibiting caspase-9-dependent apoptotic pathway.

## Background

Epstein-Barr virus (EBV), a γ-herpesvirus, causes asymptomatic infection in about 95% adults in the world [1]. EBV sets up two types of infected situation in host cells, including latent infection and lytic infection [1]. EBV replication is found in both B cells and epithelial cells. The potential oncogenic effect of EBV makes normal cell cancerization and eventually results in multiple human malignancies such as Hodgkin’s lymphoma, Burkitt’s lymphoma, nasopharyngeal carcinoma (NPC), and gastric carcinoma.

Epstein-Barr nuclear antigen 1 (EBNA1) is the only protein expressed in all three types of latent infection. Through binding to the EBV latent origin of replication (*Ori*P) [2], EBNA1 regulates EBV’s DNA synthesis and the sequential partitioning of newly synthesized viral plasmids to daughter cells [3, 4]. EBNA1 also plays an important role as an active transcription factor within viral and cellular proteins expression. EBNA1 effectively increases the activation of the family repeats element in *Ori*P and promotes the expressions of latent genes of EBNAs and latent membrane proteins (LMPs) [5, 6]. Moreover, EBNA1 can contribute to activator protein 1(AP-1) activity by inducing the transcription of the AP-1 subunits c-Jun and activating transcription factor (ATF2) [7], but decreases nuclear factor kappa B (NF-κB) activity through inhibiting the phosphorylation of the I-kappaB kinase (IKK) α/β kinase complex [8]. By binding to herpesvirus-associated ubiquitin-specific protease(USP7/HAUSP) [9] or protein kinase-casein kinase 2(CK2) [10], EBNA1 is considered to cause the destabilization of p53 [11] or promyelocytic leukemia(PML) proteins in EBV-positive cells. In addition, EBNA1 can assist malignance cells to inhibit apoptosis induced by extrinsic DNA damage drugs through destabilizing both p53 and PML proteins [10, 11].

Triptolide, a diterpene epoxide of *Tripterygium* extracts, has been demonstrated to perform a bioactive spectrum of anti-inflammatory, immunosuppressive, anti-fertility, anti-cystogenesis, and anti-cancer activities [12]. Studies also reported that triptolide could effectively kill cancer cells originated from different human organizations, including gastric [13], pancreas [14–16], brain [17], colon [18], prostate [19], blood [20], breast [21, 22], and ovary [23]. It has been reported that triptolide can stimulate the activities of caspase-8, caspase-9, and caspase-3, cleave downstream PARP and activate apoptosis [24, 25]. Caspase-9-dependent mitochondrial apoptosis pathway, rather thancaspase-8- dependent pathway, has been demonstrated as the primary way of triptolide-induced cell death [12, 24]. Triptolide can covalently bind to the subunit of the transcription factor TFIIH-XPB and inhibit its downstream gene transcription [26]. Triptolide decreases the expression of O-GlcNac transferase to influence the distribution of transcription factor specificity protein 1 (SP1) from the nucleus to cytoplasm in pancreatic tumor cells [13, 16]. Triptolide also exerts a more powerful effect against leukemia when compared with adriamycin and aclacinomycin in the clinical trial [12]. Our previous studies have indicated that triptolide could kill EBV-positive B cell lymphoma by targeting a viral oncologic protein, the latent membrane protein 1 [27]. In addition, our another study also indicated that triptolide reduced viral titers of another γ-herpesvirus, Kaposi’s sarcoma-associated herpesvirus (KSHV), by decreasing expression of latency-associated nuclear antigen 1 (LANA1) [28].

In this present study, our results indicated that triptolide inhibited the proliferation of EBV-positive NPC cells, which mainly targeted in inducing EBNA1 degradation and NPC cells apoptosis in a caspase-9-dependent pathway. Importantly, EBNA1 was critical for NPC cells to resist caspase-9-dependent apoptosis induced by triptolide. Finally, we revealed that triptolide significantly inhibited the growth of xenografted tumor induced by HONE1-Akata cell in BALB/c nude mice.

## Methods

### Cell lines and reagents

EBV-positive NPC cell lines (HONE1/Akata, HK1/Akata, and C666–1) were kindly provided by Professor S.W. Tsao (The University of Hong Kong, Hong Kong, China). An EBV-negative NPC cell line, CNE1, was kindly given by Professor. Ya Cao (The University of Zhongnan, Chang Sha, China). Human renal embryonic 293 T cells were obtained from Professor. Zhanqiu Yang (Wuhan University, Wuhan, China).HeLa cells were kindly given by Professor Hui Li (Wuhan University, Wuhan, China). All cell lines were cultured at 37 °C with a humidified atmosphere of 5% CO_2_ in growth RPMI-1640 media (Hyclone, USA) supplemented with 10% fetal bovine serum (FBS) (Gibco, USA). G418 (400 ng/ml) was additionally added into the medium of HONE1/Akata and HK1/Akata cells to maintain the stability of the recombinant EBV genomes. HeLa and 293 T cells were cultured in DMEM (Hyclone, USA) containing 10% FBS. Triptolide (Sigma, St. Louis, MO, USA), MG-132, 3-MA (Calbiochem, Billerica, MA, USA), cycloheximide (CHX) (Sigma, USA) and 12-O-tetradecanoylphorbol-13-acetate (TPA; Sigma-Aldrich) were dissolved in dimethylsulfoxide (DMSO), and were diluted to working concentration with PBS before use. Sodium butyrate (SB; Sigma-Aldrich) was dissolved in PBS directly.

CNE1/Akata cell line was made as described below. HONE1/Akata cells were induced to the lytic form by adding TPA (40 ng/ml) and SB (3 mM) into culture medium for 48 h in order to produce virions. The cell culture medium was collected. After centrifugation at 2000 rpm for 5 min, the supernatant containing virions was used to infected CNE1 cells. At 24 h post-infection, G418 was added into the medium to get a concentration of 1000 ng/ml. After 24 h, uninfected cells would die. The living cells were continuously cultured in medium containing G418 (400 ng/ml). After stably growing for 5 generations, CNA1/Akata cells were permitted for further experiments.

### Cell viability assay

HONE1/Akata, HK1/Akata, C666–1, and CNE1 cells (1 × 10^4^ cells/well) were placed in 96-well plates and treated with DMSO control (0.01%) or increasing concentrations of triptolide (25, 50, 100, or 200 nM) for 24 and 48 h. Ten microliters of the Cell Counting Kit-8 (DOJINDO, Tokyo, Japan) reagent were then added to each well and the plates were incubated at 37 °C for 1 h in dark. The optical density (OD) value was detected at an absorbance of 450 nm using an ELx800 microimmunoanalyser (BioTek Instruments, Inc., Winooski, VT, USA).

### Colony-formation assay

HONE1/Akata, HK1/Akata, C666–1, and CNE1 cells were placed in 35 mm culture dishes (500 cells/dish) and cultured in standard medium with DMSO control (0.01%) or triptolide (1, 2, or 5 nM) for 2 weeks. Colony formation units were stained with 0.5% (*w*/*v*) crystal violet prepared in 0.6% (*v*/v) glutaraldehyde solution for 1 min, and then cell colony in culture dishes were photographed.

### Cell cycle analysis

HONE1/Akata and HK1/Akata cells were seeded in 6-well plates and treated with DMSO control (0.01%) or triptolide (50 nM) for 24 h. NPC cells were digested with 0.25% EDTA-trypsin (Gbico, USA), and 2 × 10^5^ cells were washed 2 times with cold PBS solution, and then resuspended with precooled75% ethanol. After preserved at − 20 °C for 24 h, cells were centrifuged at 1000 rpm for 5 min and resuspended in 0.5 ml cold PBS. Cells were mixed with reagent A (Multisciences, ShangHai, China). Following incubation in dark at 4 °C for 30 min, the whole cells were analyzed immediately by a Beckman-Coulter system (EPICS Altra II; BeckmanCoulter, Fullerton, CA, USA).

### Apoptosis analysis

The apoptosis levels ofHONE1/Akata and HK1/Akatacells were evaluated with Annexin V-FITC/7-AAD apoptosis detection kit (Multisciences, Shanghai, China) as manufacturer described. Briefly, cells were placed in 6-well plates and treated with vehicle control (0.01% DMSO) or triptolide (100 or 200 nM) for 24 h. Cells were digested and 2 × 10^5^ cells were washed 3 times with PBS and resuspended in 500 μl of 1× binding buffer, followed by adding 5 μl of Annexin V-FITC and 10 μl of 7-AAD, and incubating in dark at room temperature for 30 min. The whole cells were analyzed immediately by a Beckman-Coulter system (EPICS Altra II; BeckmanCoulter, Fullerton, CA, USA).

### Cell transfection

Plasmid pSG5-EBNA1 (P-ala) containing the full-length EBNA1 was constructed by Neuron Biotech Corporation (Shanghai, China). PGEM-EBNA1 (V-val) was a gift from Professor Yixin Zeng (Sun Yat-Sen University, Guangzhou, P.R. China) and described in previous study [29]. The plasmids were isolated using the plasmid DNA extraction kit (Cat No. CFLKP001–50, Chunfenglv Biomedical Technology, Beijing, P. R. China) according to the manufacturer’s instructions.

EBNA1 siRNA was designed according to the previous study, and the siRNA target DNA sequence of EBNA1 was 5’-GGACTACCGACGAAGGAAC-3′ [30]. The sequence was synthesized by GenePharma (Suzhou, China). For transfection, cells were placed in 6-well plates with 4 × 10^5^cells per well. The cells were transiently transfected by using X-tremeGENE HP DNA Transfection Reagent (Roche, Basel, Switzerland) with pSG5 (the empty vector) or pSG5-EBNA1 as indicated. At 4 h posttransfection, cells were washed with PBS and treated with DMSO (0.01%) or triptolide. After 24or 48 h incubation, cells were prepared for Western blot assay or real-time PCR.

### RNA isolation, reverse transcription, and real-time PCR

TRIzol Reagent (Life, USA) was used to extract the total RNA according to the manufacturer’s instructions. The concentration of RNA was determined by Nanodrop 2000 (Thermo, USA). RNAs (1 μg) were reverse transcribedinto cDNA using Reverse Transcription kit (Takara, Tokyo, Japan) as the manufacturers’ instructions. Expression levels of EBNA1 mRNAs were quantified by the CFX96 Real-Time PCR Detection System using an SYBR Premix Ex Taq kit (Takara, Tokyo, Japan). The primers were as follows: EBNA1 forward, 5’-TCATCATCATCCGGGTCTCC-3′; EBNA1 reverse, 5’-CCTACAGGGTGGAAAAATGGC-3’;GAPDH forward, 5’-GGTGGCTTCTGACTTCAACA-3′; GAPDH reverse, 5’-GTTGCTGTAGCCAAATTCGTTGT-3′. The EBNA1 levels were normalized to the housekeeping gene GAPDH. All experiments were repeated independently 3 times.

### Western blot assay

Cells treated in different conditions were harvested in RIPA lysis buffer (BeyotimeInstitute of Biotechnology, Shanghai, China) supplemented with 0.5% cocktail protease inhibitor (Roche) and 0.5 mMphenylmethylsulfonyl fluoride (PMSF). Followed by storing on ice for 10 min, the cell lysates were collected and sonicated for 15 s. After centrifugation at 12,000 x g for 15 min, the supernatants were collected and transferred into new tubes, and the protein concentration was measured by BCA protein assay (BioRad, USA). Equal amounts of proteins were then mixed with 5× loading buffer (250 nMTris-Hcl (pH 6.8), 0.5% BPB, 50% glycerol, 10% SDS, 5% β-mercaptoethanol) and boiled for 5 min. Then the mixtures were subjected to 10% SDS-PAGE gels in running buffer and subjected to immunoblot analyses.

The primary antibodies used were as follows: GAPDH (Cat No. 10494–1-AP, 1:5000; Proteintech, Wuhan, China); β-actin (Cat No. CFLKT001, 1:10,000, Beijing Chunfenglv Biomedical Technology Co., Ltd., Beijing, China); caspase-3 (Cat No. 9668, 1:1000; Cell Signaling Technology, USA); cleaved caspase-3 (Cat No. 9664, 1:1000; Cell Signaling Technology, USA); caspase-9 polyclonal antibody (Cat No. A2636, 1:1000; ABclonal, Boston, UK); cleaved PARP-1(Cat No. sc-56,196, 1:500; Santa Cruz Biotechnology, USA); P53 (DO-1) (Cat No. sc-126, 1:500; Santa Cruz Biotechnology, USA); Lamin-A (Cat No. 4777; 1:2000, Cell Signaling Technology, USA); Beta-Tublin (Cat No. 4777; 1:3000, Cell Signaling Technology, USA). Western blot gray values were determined by ImageJ software (National Institutes of Health, USA).

### Hoechst 33,258 staining

Cell nuclear fragmentation was examined by Hoechst 33,258 (Beyotime Biotechnology, China). HONE1/Akata cells (4 × 10^5^) were placed in 6-well plate with a glass slide in the well, then treated with DMSO control (0.01%) or triptolide (100 nM) for 24 h. Cells were fixed for 30 min at room temperature by using 4% paraformaldehyde, then washed 3 times with PBS. Followed by permeabilization with 0.1% TritonX-100 in PBS at room temperature for 15 min, cells were then washed 3 times with PBS. Then, cells were blocked with 5% bovine serum albumin (BSA) in PBS for 30 min and washed 3 times with PBS. Cells were stained with Hoechst 33,258 in dark for 5 min, and washed 3 times with PBS. The slides were covered with glycerinum and observed using a fluorescence microscope.

### Peripheral blood mononuclear cell (PBMC) isolation

Patient samples were obtained from Clinical Laboratory of Renmin Hospital of Wuhan University. Patients have been informed of the contents of the assay. All the selected patients were detected to carry EBV in plasma and were confirmed as EBV-positive patients. PBMC was isolated from whole blood by Ficoll-Hypaque density gradient centrifugation according to manufacturer’s instructions (Sigma, USA) and cultured in RPMI 1640 containing 10% FBS (Hyclone, USA), 1% glutamine (Sigma, USA), and 1% penicillin/streptomycin (Sigma, USA).

### Immunohistochemistry

Immunohistochemistry was performed using antibodies against EBNA1 or cleaved caspase-3 under the manufacturer’s instructions. The tissue sections were fixed with 10% neutral buffered formalin for 12 h. After dehydration, the tissue sections were paraffin-embedded. The paraffin section was deparaffinized using xylene for 20 min and then permeated in gradient concentrations of alcohol. For antigen retrieval, the tissue section was boiled in citric acid (pH 6.0) for 20 min. Endogenous peroxidase activity was quenched with 3% H_2_O_2_ for 10 min. Then the slide was blocked in goat serum for 1 h at room temperature, followed by incubating with primary antibodies (EBNA1, Cat No. 8329, 1:1000, Abcam, USA; cleaved caspase-3, Cat No. 9664, 1:1000; Cell Signaling Technology, USA) overnight at 4 °C. After incubated for 15 min at room temperature with secondary antibody (MaxVision™ Kits, MXB, China) conjugated with horseradish peroxidase-labeled polymer, tissue section was incubated for 1 min with diaminobenzidine. The section was counterstained with hematoxylin lightly.

### Immunofluorescence assay

The pre-process of immunofluorescence assay was performed as described in the part of immunohistochemistry. After incubation with primary antibody (EBNA1, Cat No. 8329,1:1000,Abcam, USA) for 30 min at room temperature, the slides were incubated for 50 min at 37 °C with the Alexa Fluor 488-conjugatedsecondary antibody (goat anti-mouse, cat. no. A32723, 1:100, Invitrogen, USA). The morphology of cell nuclei was observed by DAPI staining. Images were photographed and synthesized by MicroPublisher (Q-IMAGING, Canada).

### Animal studies

HONE1/Akata cells (1 × 10^7^) were inoculated subcutaneously into both flanks of 4-week-old male BALB/c nude mice (purchased from the ABSL-3 animal lab at Wuhan University). Seven days later, when tumor became to be palpable, mice (five per group) were treated with a single intraperitoneal injection of triptolide at 0.4 mg/kg or DMSO daily. After 21 days of treatment, mice were sacrificed by cervical dislocation. Mice weights and tumor sizes were measured, and tumor volume was calculated as 0.5 × length × width× width. Tissue samples were collected and fixed in 10% neutral buffered formalin, embedded, and sectioned at a 5 μm of thickness.

### Nuclear and cytoplasmic protein extraction

HONE1/Akata cells (1 × 10^6^) were placed in 6 cm plates and cultured for 48 h. Then the cells were subjected to extraction of nuclear and cytoplasmic proteins by NE-PER Nuclear and Cytoplasmic Extraction Reagents (Thermo, USA). The detailed process of extraction was performed according to the introduction of manufacturer. After protein concentration was determined, the nuclear and cytoplasmic proteins were mixed with 5× loading buffer and boiled for 5 min, and subjected to Western blot assay.

### Immunohistochemistry analysis of EBNA1 and Ki67 expression in NPC biopsies

All the NPC biopsies and correlative clinical data were obtained from the department of pathology, Renmin Hospital of Wuhan University. Expression of EBNA1 and Ki67 was analyzed by two pathologists, Dr. Heng Zhou and Dr. Wen Liu (department of pathology, Renmin Hospital of Wuhan University).

### Statistical analysis

Data were shown as the mean ± standard deviation (mean ± SD) and analyzed by Student’s t-test using GraphPad Prism for Windows version 5.0 (GraphPad Software, La Jolla, USA), and *p*-values < 0.05 were considered as statistically significant.

## Results

### Triptolide effectively suppresses proliferation of NPC cells

**Fig. 1:**
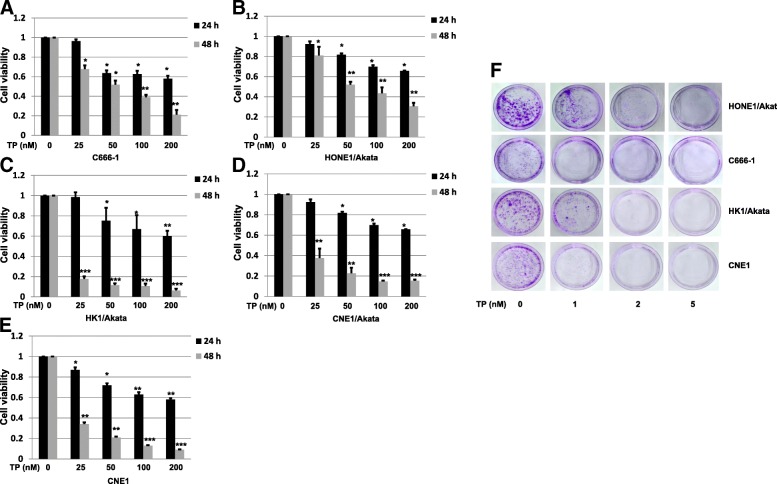
Triptolide (TP) suppresses proliferation of EBV-positive NPC cells. Cells were placed in 96-well plates, treated with DMSO control (0.01%) or TP (25, 50,100, or 200 nM) for 24 h or 48 h. Cell viability of C666–1 **a**, HONE1/Akata **b**, HK1/Akata **c**, CNE1/Akata **d**, and CNE1 (**e**) were detected by a CCK-8 kit. **f** Cells were placed in 35 mm culture dishes and then cultured in standard medium with DMSO control (0.01%) or TP (1, 2, 5 nM). After 2 weeks, the colony-formation analysis was performed (*: *P <* 0.05; **: *P* < 0.01; ***: *P* < 0.001)

To determine whether triptolide decreases cell viabilities of NPC cells, 5 kinds of NPC cells were treated with triptolide for 24 h or 48 h in a dose-dependent manner. As shown in Fig. 1a–e, triptolide significantly inhibited cell viabilities of C666–1, HONE1/Akata, HK1/Akata, CNE1/Akata and CNE1 cells in comparison with the cells treated with DMSO control (0.01%). The IC_50_ of C666–1, HONE1/Akata, HK1/Akata, CNE1/Akata and CNE1 were 55.43, 76.56, 1.12, 11.04 and 10.66 nM, respectively. To further confirm the above results, colony formation assay was performed. Four kinds of EBV-positive NPC cells were placed in 35 mm culture dishes, treated with DMSO control (0.01%) or triptolide (1, 2, or 5 nM) for 2 weeks, and stained with crystal violet. As shown in Fig. 1f, low concentration of triptolide remarkably reduced colony formation at both colony size and numbers. These results strongly suggest triptolide effectively inhibits proliferations of both EBV-negative and EBV-positive NPC cells.

### Triptolide arrests cell cycles in S phase

**Fig. 2:**
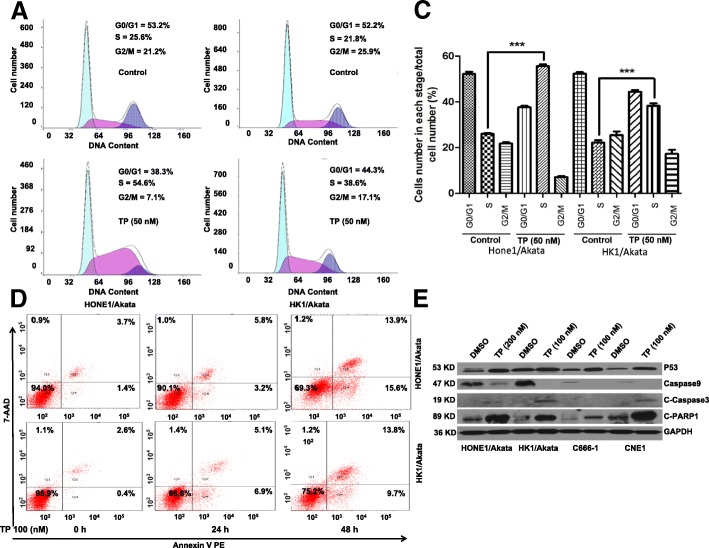
TP arrests cell cycles and induces apoptosis in EBV-positive NPC cells. **a** HONE1/Akata and (**b**) HK1/Akata cells were treated with DMSO control (0.01%) or TP (50 nM) for 24 h. The distribution of cell cycle was determined by flow cytometry. **c** The cell cycle distribution is quantified by the bar graphs. **d** HONE1/Akata and HK1/Akata cells were treated with DMSO control (0.01%) or TP (100 nM) for 24 hand 48 h, respectively. Apoptosis assay was performed by flow cytometry. **e** Four kinds of NPC cells were treated with DMSO control (0.01%) or TP for 48 h, protein expression was determined by Western blotting assay. (*: *P <* 0.05; **: *P* < 0.01; ***: *P* < 0.001)

To determine whether triptolide affects cell cycles of NPC cells, HONE1/Akata and HK1/Akata cells were treated with DMSO control (0.01%) or triptolide (50 nM) for 24 h and then harvested. The cell cycle distributions were analyzed by flow cytometry (Fig. 2a, b). As shown in Fig. 2c, the cell cycles of HONE1/Akata and HK1/Akata were all arrested in S phase. The fractions of S phase cells of HONE1/Akata and HK1/Akata were increased by29.0% and 16.8% respectively when treated with triptolide (50 nM). These results reveal that triptolide arrests cell cycles in S phase effectively.

### Triptolide induces mitochondrial apoptosis in NPC cells

To examine the apoptotic effect induced by triptolide, NPC cells were treated with DMSO control (0.01%) or triptolide (100 nM) for 24 h or 48 h, respectively. As shown in Fig. 2d, treatment of HONE1/Akata and HK1/Akata cells with triptolide resulted in apoptosis in a time-dependent manner. In addition, western blotting was performed to detect the expression of several apoptosis-related proteins. As shown in Fig. 2e, triptolide decreased expression of caspase-9 and increased expression of p53, cleaved caspase-3 and cleaved PARP-1 in EBV-positive HONE1/Akata, HK1/Akata, C666–1 and EBV-negative NPC CNE1cells. All these results indicate that triptolide induces apoptosis of NPC cells.

### Triptolide decreases expression of EBNA1 in NPC cell lines

**Fig. 3:**
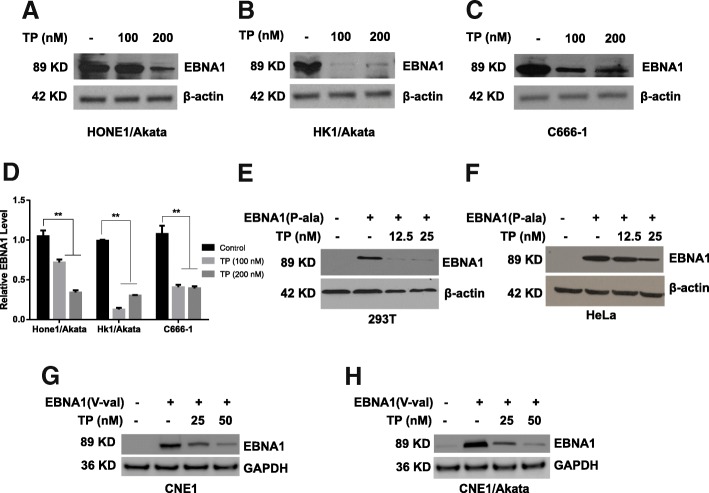
TP decreases the expression of EBNA1. **a** HONE1/Akata, **b** HK1/Akata, and (**c**) C666–1 cells were treated with DMSO (0.01%) or TP (100 or 200 nM) for 48 h, respectively. Fig. **d** showed the quantified bar graph of EBNA1 expression in NPC cell lines treated with TP. **e** 293 T or (**f**) HeLa cells were transfected with pSG5 or pSG5-EBNA1(P-ala) for 4 h, followed by treatment with TP (12.5 or 25 nM) for 44 h. **g** CNE1 and (**h**) CNE1/Akata were transfected with pGEM or pGEM-EBNA1(V-val) for 4 h, followed by 44 h treatment with TP (25 or 50 nM). Whole-cell extracts were prepared and subjected to Western blot analysis

Our previous work demonstrated that triptolide decreases LANA1 expression in KSHV-positive primary effusion lymphoma [28]. Therefore, we next wanted to examine whether triptolide affects the expression of EBNA1 in EBV-positive NPC cells. Three different EBV-positive NPC cell lines (HONE1/Akata, HK1/Akata, and C666–1) were treated with DMSO control (0.01%) or triptolide (100 or 200 nM) for 48 h. As shown in Fig. 3a–d, the expression of EBNA1 in HONE1/Akata, HK1/Akata, and C666–1 was significantly decreased by 68.21%, 70.09% and 65.37% when treated with 200 nM triptolide. After that, we detected whether triptolide affects the transient expression of EBNA1, which is independent of EBV genome. PSG5-EBNA1(P-ala) plasmids were transfected into EBV-negative 293 T and HeLa cells, and EBNA1(V-val) plasmids were transfected into CNE1 and CNE1/Akata cells for 4 h, then cells were treated with triptolide for another 44 h. As shown in Fig. 3e–h, triptolide significantly decreased the expressions of EBNA1(P-ala) and EBNA1(V-val). All these results suggest that triptolide decreases EBNA1 expression.

### Triptolide reduces stability and half-life of EBNA1 and induces proteasomal degradation of EBNA1

**Fig. 4:**
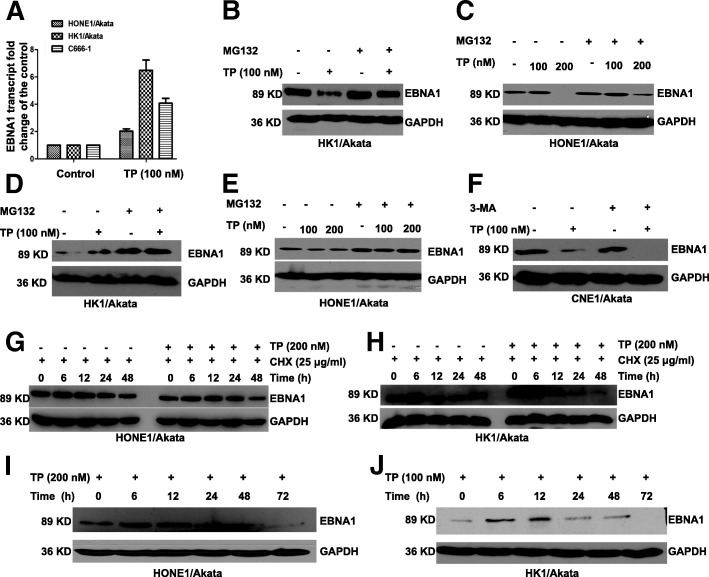
TP reduces the half-life of EBNA1 and induces proteasomal degradation of EBNA1. **a** HONE1/Akata, HK1/Akata, and C666–1 cells were treated with DMSO (0.01%) or TP (100 nM) for 24 h. Transcriptional levels of EBNA1 were examined by the real-time PCR. **b** HK1/Akata and (**c**) HONE1/Akata cells were treated for 48 h with DMSO control (0.01%) or TP (100 nM) in the absence or presence of proteasome inhibitor MG-132 (50 μM) for the last 12 h. **d** HK1/Akata and (**e**) HONE1/Akata cells were treated with DMSO control (0.01%) or TP (100 nM) in the absence or presence of proteasome inhibitor MG-132 (50 μM) for 24 h. **f** CNE1/Akata cells were treated with DMSO control (0.01%) or TP (100 nM) in the absence or presence of the autophagy inhibitor 3-MA (10 mM). **g** HONE1/Akata and (**h**) HK1/Akata cells were treated with DMSO (0.01%) or TP (200 nM) in the presence of CHX (25 μg/ml) for indicated times. **i** HONE1/Akata and (**j**) HK1/Akata cells were treated with 0.01% DMSO or TP for 6, 12, 24, 48, and 72 h, respectively. Whole-cell extracts were prepared and subjected to Western blot analysis

The decrease of protein levels may be due to the suppression of mRNA levels, so we next detected the effects of triptolide on the transcriptional levels of EBNA1. HONE1/Akata, HK1/Akata, and C666–1 cells were treated with 0.01% DMSO control or triptolide (100 nM) for 24 h. As shown in Fig. 4a, interestingly, triptolide increased EBNA1 transcriptional levels to varying degrees. Since triptolide increased EBNA1 mRNA levels, we wondered whether triptolide decreases protein stability or posttranslational modification of EBNA1. To determine whether triptolide affects proteasome pathway in EBV-positive NPC cells, HK1/Akata and HONE1/Akata were treated with DMSO control (0.01%) or triptolide (100 or 200 nM) for 48 h in the absence or presence of proteasome inhibitor MG-132 (50 μM) for 12 h. In addition, HK1/Akata and HONE1/Akata were treated with DMSO control (0.01%) or triptolide (100 or 200 nM) for 24 h in the absence or presence of proteasome inhibitor MG-132 (50 μM) for 24 h. Cells were harvested and the expression of EBNA1 was analyzed by western blotting. As shown in Fig. 4b, c, MG132 attenuated triptolide-induced EBNA1 degradation in HK1/Akata and HONE1/Akata cells. As shown in Fig. 4d, e, MG132 increased EBNA1 expression in the presence or absence of triptolide both in HK1/Akata and HONE1/Akata cells. To determine whether triptolide induces EBNA1 degradation through autophagy pathway, CNE1/Akata cells were treated with triptolide (100 nM) in the presence or absence of 3-MA (10 mM). As shown in Fig. 4f 3-MA did not attenuate the inhibitory effect of triptolide on the expression level of EBNA1 in CNE1/Akata cells. These results suggested that triptolide induces the degradation of EBNA1 through a proteasome-ubiquitin pathway.

We then supposed whether triptolide affects EBNA1 stability. Hence, we used CHX as an adjuvant to investigate it. HONE1/Akata and HK1/Akata cells were treated with DMSO (0.01%) or triptolide (100 nM) in the presence of CHX (25 μg/ml) in a time-dependent manner. As shown in Fig. 4g, h, the group treated with both CHX and triptolide accelerated degradation of EBNA1 in comparison with the group treated with CHX alone, which indicated that triptolide reduces the half-life of EBNA1 and decreases its stability directly. In addition, the expression of EBNA1 after triptolide treatment over time was tested. As shown in Fig. 4i, j, when treated with triptolide, EBNA1 protein levels were increased at 12 h in HONE1/Akata and HK1/Akata, and then decreased with the passage of time. All these results show that triptolide inhibits EBNA1 expression through impairing the stability of EBNA1 and inducing proteasomal degradation of EBNA1.

### Exogenous EBNA1 attenuates the triptolide-induced mitochondrial apoptosis

**Fig. 5:**
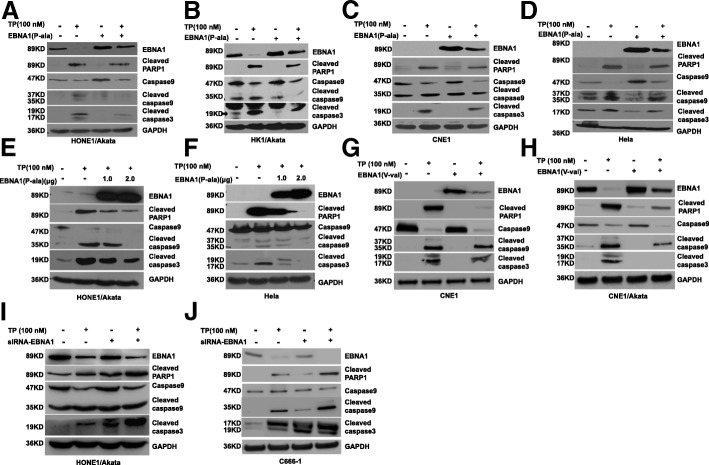
Over-expression of EBNA1 attenuates mitochondrial apoptosis induced by TP. **a** HONE1/Akata, **b** HK1/Akata, **c** CNE1, and (**d**) HeLa cells were transfected with pSG5-EBNA1(P-ala) for 4 h, followed by treatment with DMSO control (0.01%) or TP(100 nM) for 44 h, respectively. **e** HONE1/Akata and (**f**) Hela cells were transfected with pSG5-EBNA1(P-ala) at indicated concentrations for 4 h, followed by treatment with DMSO control (0.01%) or TP(100 nM) for 44 h, respectively. **g** CNE1 and (**h**) CNE/Akata cells were transfected with pGEM-EBNA1(V-val) for 4 h, then treated with DMSO control (0.01%) or TP (100 nM) for 44 h, respectively. **i** HONE1/Akata and (**j**) C666–1cells were transfected with EBNA1 siRNA for 4 h, then treated with DMSO control (0.01%) or TP (100 nM), respectively. Whole-cell extracts were prepared and subjected to Western blot analysis

EBNA1 plays an anti-apoptosis role acting on USP7/HAUSP and CK2 pathways [9–11], thus we wondered whether EBNA1 could inhibit apoptosis effects induced by triptolide in NPC cells. Briefly, pSG5-EBNA1(P-ala) plasmids were transfected into HONE1/Akata, HK1/Akata and CNE1 cells for 4 h, then treated with triptolide (100 nM) for another 44 h. As shown in Fig. 5a, b, over-expression of EBNA1 attenuated the expression of several apoptosis-related proteins, including cleaved PARP-1, cleaved caspase-3, and cleaved caspase-9. To determine whether EBNA1 attenuates apoptotic effect outside the context of the EBV genome, the above experiments were repeated in an EBV-negative NPC cell line-CNE1 and human epithelial cervical cancer HeLa cells. Similar results were obtained and shown in Fig. 5c, d.

In addition, dose-dependent pSG5-EBNA1 (P-ala) transfections in HONE1/Akata and HeLa cells were complemented to confirm the anti-apoptosis effect of EBNA1. As shown in Fig. 5e, f, EBNA1 possessed the biological property to restrain apoptosis in a dose-dependent manner in HONE1/Akata and HeLa cells. EBNA1 (V-val), the dominant subtype in Asian [31, 32], was reported to be found in tumor tissues of Asian NPC patients. Hence, pGEM-EBNA1(V-val) or control plasmid was transfected into CNE1 and CNE1/Akata cells in the presence or absence of triptolide (100 nM). Over-expression of EBNA1 (V-val) significantly attenuated mitochondrial apoptosis-associated active protein expressions, including cleaved PARP-1, cleaved caspase-3, and cleaved caspase-9 (Fig. 5g, h).

Furthermore, EBNA1 was knockdown to evaluate its anti-apoptosis effects. EBNA1 siRNA or control siRNA was respectively transfected into HONE1/Akata or C666–1cells. After 24 h transfection, cells were then treated with DMSO (0.01%) or triptolide (100 nM) for 48 h. As shown in Fig. 5i, j, knockdown of EBNA1 significantly enhanced triptolide-induced caspase-9 apoptotic pathway. These results suggest that EBNA1 could inhibit triptolide-induced mitochondrial apoptosis in NPC cells.

### Exogenous EBNA1 attenuates the inhibitory and apoptotic effects of triptolide

**Fig. 6:**
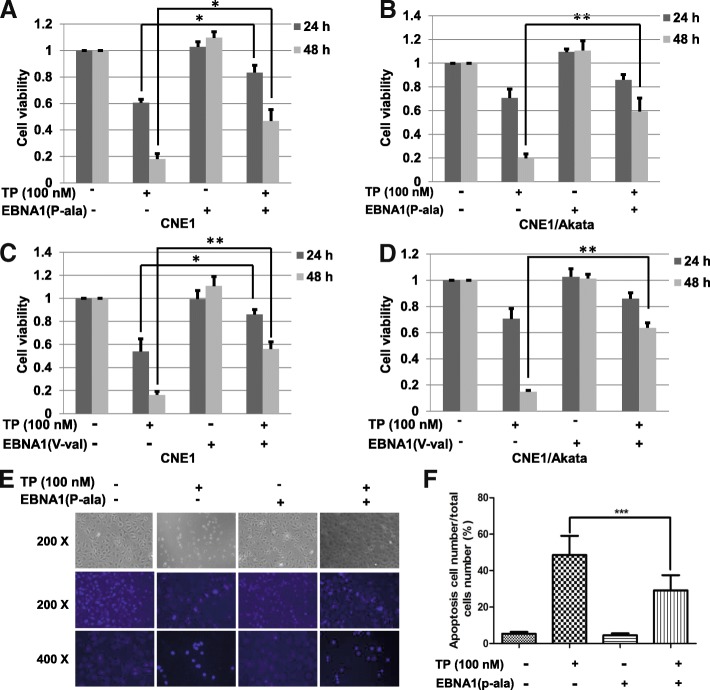
Over-expression of EBNA1 attenuates TP-induced cell killing and apoptotic effect. **a** CNE1 and (**b**) CNE1/Akata cells were transfected with pSG5-EBNA1(P-ala) or pSG5 for 4 h, and treated with TP (100 nM) for 24 h or 48 h. **c** CNE1 and (**d**) CNE1/Akata cells were transfected with pGEM-EBNA1(V-val) or pGEM for 4 h, and treated with TP (100 nM) for 24 h or 48 h. Cell viability of NPC cells were detected by a CCK-8 assay. **e** HONE1/Akata cells were treated with DMSO control (0.01%) or TP (100 nM) for 24 h. After staining with Hoechst 33,258, cells were observed using a fluorescence microscope. **f** The apoptosis cell numbers were calculated, quantified, and shown by the bar graphs (*: *P <* 0.05; **: *P* < 0.01; ***: *P* < 0.001)

Previous studies have suggested that targeting on EBNA1 is an effective strategy of NPC therapy [33–35]. To determine if EBNA1 reduction contributes to the killing effect of triptolide, CNE1 and CNE1/Akata cells were transfected with control plasmids or EBNA1 plasmids (P-ala or V-val) for 4 h, and then treated with triptolide (100 nM) for 24 or 48 h. The CCK-8 assay was used to test the cell viability. As shown in Fig. 6a–d, over-expression of EBNA1 (P-ala or V-val) attenuated the inhibitory of triptolide significantly. These results suggest that decreased EBNA1 expression substantially contributes to the killing effect of triptolide on NPC cells.

To further verify the anti-apoptotic effect of EBNA1, Hoechst staining was used to observe anti-apoptosis effects of EBNA1 in HONE1/Akata cells (Fig. 6e). As shown in Fig. 6f, the average apoptotic rate degraded from 48.8% in the group of treatment with triptolide alone to 29.5% in the group of transfection with pSG5-EBNA1. Above all, these results may indicate that EBV attenuates triptolide-induced NPC cells death through the anti-apoptosis effect of EBNA1.

### Low-toxicity of triptolide decreases the expression of EBNA1 in PBMCs

**Fig. 7:**
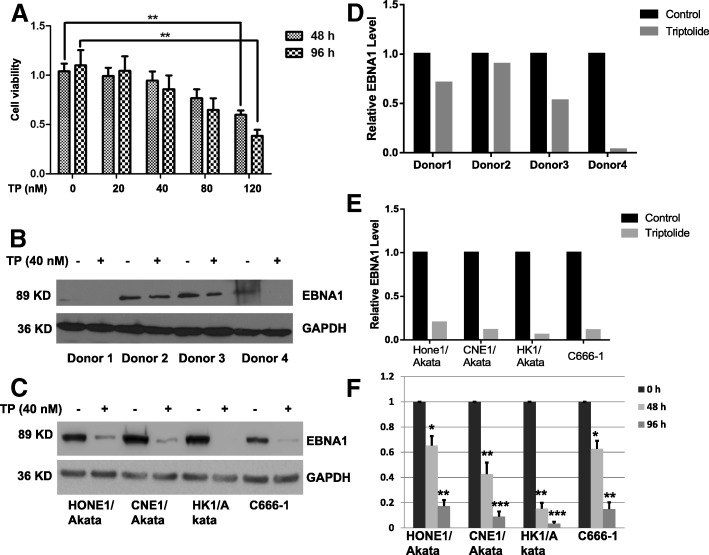
TP at a non-toxic concentration decreases EBNA1 expression in PBMCs and NPC cells. PBMCs were isolated from EBV-positive donors and incubated with DMSO (0.01%)or dose-dependent TP for 96 h. **a** Cell viability of PBMCs was detected by a CCK-8 assay. **b** PBMCs were treated with DMSO (0.01%) or TP (40 nM) for 96 h. **c** HONE1/Akata, CNE1/Akata, HK1/Akata, and C666–1 cells were treated with DMSO (0.01%) or TP (40 nM) for 96 h. Whole-cell extracts were prepared and subjected to Western blot analysis. Quantified analysis of EBNA1 expression in PBMCs (**d**) or NPC cell lines (**e**) were performed by Image J. **f** HONE1/Akata, CNE1/Akata, HK1/Akata and C666–1 cells were treated with DMSO (0.01%) or TP (40 nM) for 0, 48, and 96 h. Cell viability of NPC cells were detected by a CCK-8 assay (*: *P <* 0.05; **: *P* < 0.01; ***: *P* < 0.001)

To examine whether a safe concentration of triptolide decreases the expression of EBNA1, PBMCs were cultured in 96-well plate and treated with a dose-dependent triptolide (0, 20, 40, 80 and 120 nM) for 96 h. As shown in Fig. 7a, triptolide significantly inhibited cell viability of PBMCs at 120 nM. Hence, a safe and low-toxicity concentration of 40 nM was selected to determine if triptolide decreased expression of EBNA1. PBMCs were treated with 40 nM triptolide for 96 h and then harvested for Western blotting. As shown in Fig. 7b, d, expression of EBNA1was decreased significantly in different donors. To determine whether the safe concentration of triptolide decreases the expression of EBNA1 in NPC cells, HONE1/Akata, CNE1/Akata, CNE1/Akata, and C666–1 cells were treated with 40 nM triptolide for 96 h. Western blotting showed that the expression of EBNA1 was decreased significantly in all NPC cell lines (Fig. 7c, e). In addition, cells treated with 40 nM of triptolide significantly inhibited the cell viability of NPC cells at both 48 h and 96 h (Fig. 7f). These results indicate that the safe concentration of triptolide reduces EBNA1 expression in EBV positive cells.

### Triptolide inhibits proliferation of NPC cell-induced tumor in vivo

**Fig. 8:**
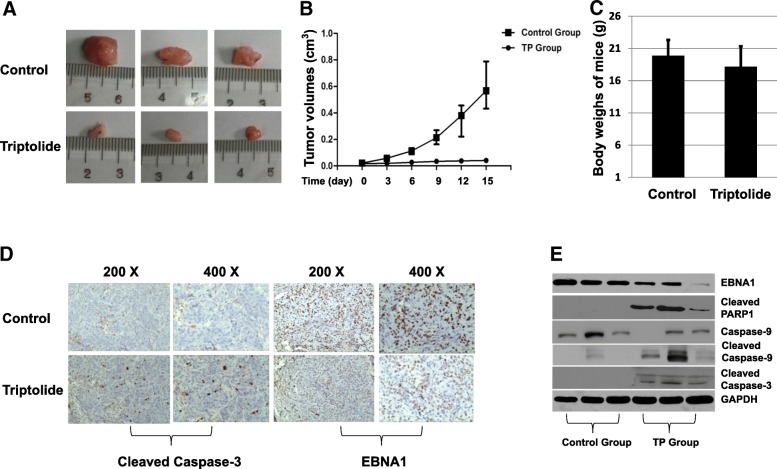
TP inhibits proliferation of NPC cell-induced tumor in vivo. BALB/c nude mice with the HONE1/Akata cell-induced tumors were treated with DMSO (0.01%) or 400 μg/kg/day triptolide (*n* = 6 animals per group) for 21 days. **a** Representative photos of the dissected tumors were shown. **b** Tumor volumes and (**c**) mice weight were given. **d** The expression of cleaved caspase-3 and EBNA1 in tumor tissues were performed by immunohistochemical assay. **e** The expression of EBNA1, cleaved PARP-1, caspase-9, cleaved caspase-9, and cleaved caspase-3 in control and TP groups were detected by Western blot assay

Since our above results indicated that triptolide inhibits proliferation of NPC cells in vitro, effects of triptolide in vivo were also examined by using BALB/c nude mice. To study whether triptolide influences growth of NPC in BALB/c mice, 1 × 10^7^ HONE1/Akata cells were injected subcutaneously into flanks of the mice. When tumor had grown to be palpable (7 days later), the mice were treated with 0.4 mg/kg triptolide or DMSO daily via intraperitoneal injection. After 15 days, mice were euthanized and tumors were measured and weighed. As shown in Fig. 8a–c, representative photos (8A), tumor volumes (8B) and tumor weights (8C) suggested that triptolide could inhibit the growth of tumors. To determine the mechanism how triptolide suppresses the growth of tumor, the expression and distribution of EBNA1 and cleaved caspase-3 in the tumor tissues were analyzed by immunohistochemical assay. As shown in Fig. 8d, the tissue slides of triptolide group showed stronger positive signals of cleaved caspase-3 than those of control slides. Conversely, EBNA1 positive signals in the triptolide group were weaker than the slides in the control group. In addition, western blotting assay was performed to detect expression of EBNA1 and the associated apoptotic proteins. As shown in Fig. 8e, EBNA1 expression was decreased in triptolide group compared with control group. Triptolide increased the expression of associated apoptotic proteins (cleaved PARP1, cleaved caspase-3, and cleaved caspase-9) in tumor tissues. These results suggest that triptolide inhibits proliferation of NPC induced by EBV-positive HONE1/Akata cells.

### Distribution of EBNA1 is associated with tumor growth in NPC patients

**Fig. 9:**
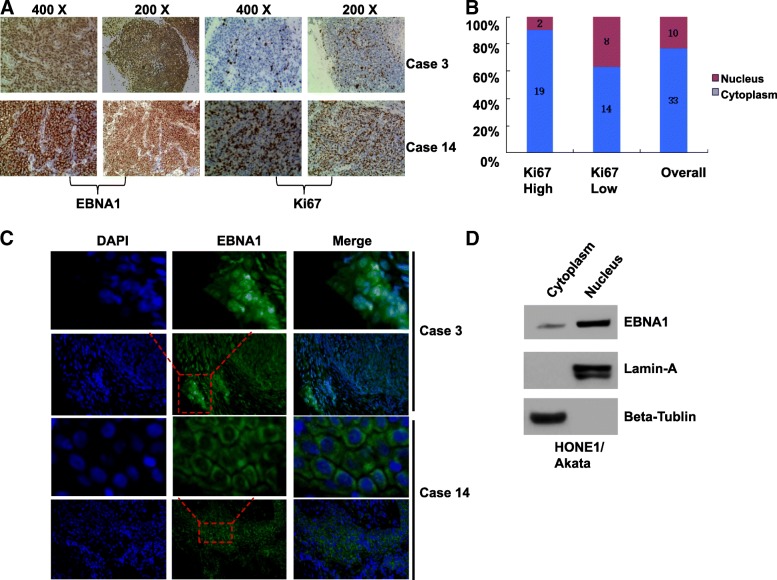
EBNA1 distribution is associated with tumor growth in NPC patients. **a** The expression of EBNA1 and Ki67 were determined by immunohistochemistry analysis in NPC biopsies from patients. **b** The distribution frequencies of EBNA1 in Ki67-low and Ki67-high group were shown. **c** The distribution of EBNA1 was determined by immunofluorescence assay in NPC biopsies from patients. **d** Nucleoprotein and cytoplasmic proteins of HONE1/Akata cells were separated and subjected to Western blotting analysis

Since the above results suggested triptolide targets EBNA1 to inhibit proliferation of EBV-positive cells and tumor growth in the animal model experiment, we continue to investigate the distribution of EBNA1 in NPC patients. Totally 43 non-keratinizing NPC biopsy tissues from patients in Renmin Hospital of Wuhan University were investigated, which included 26 undifferentiated carcinomas and 17 differentiated carcinomas (1 carcinoma/patient) (Table 1). All the chosen biopsy tissues were only EBV-positive tissues by detecting the EBV-encoded small RNA (EBER), a small RNA encoded by EBV latent infection stage (not shown). Immunofluorescence and immunohistochemistry assays were performed to detect the expression and distribution of EBNA1. Among all the EBV-positive tissues, the EBNA1 protein was expressed (100%). However, EBNA1 was expressed in the cytoplasm of all the 43 tumor biopsies but expressed in both nuclear and cytoplasm of 10 tumor biopsies in NPC cells. Results of representative cases were shown in Fig. 9a, c. Nuclear and cytoplasmic protein extraction was performed to detect EBNA1 distribution in NPC cells. As shown in Fig. 9d, the expression of EBNA1 was observed both in nucleus and cytoplasm of HONE1/Akata cells.

**Table 1:** The associations between EBNA1 distribution and sex, histopathological data, and KI67 expression

Criteria		Specimen Nos. (%)	EBNA1 Distribution (%)		χ^2^	Df	*p*
			Nucleus^a^	Cytoplasm			
Sex	Female	18 (41.9)	5 (20)	20 (80)	0.36	1	0.55
	Male	25 (58.1)	5 (27.8)	13 (72.2)			
Histological Types	Differentiated	17 (39.5)	5 (29.4)	12 (70.6)	0.6	1	0.44
	Undifferentiated	26 (60.5)	5 (19.2)	21 (80.8)			
KI67 expression	High	21 (48.8)	2 (9.5)	19 (90.5)	4.34	1	0.037^b^
	Low	22 (51.2)	8 (36.4)	14 (63.6)			

Note: ^a^ EBNA1 expression in both nucleus and cytoplasm of NPC biopsy

^b^ *p* < 0.05

The associations among sex, histological type, Ki67 expression, and EBNA1 distribution were analyzed statistically. Notably, Ki67 is a nuclear protein, which is an indicator of proliferation state of the cancer cells. Therefore, patients were divided into two groups based on the Ki67 expression. As shown in Table 1, EBNA1 distribution was independent of sex and histological type. The immunohistochemistry results revealed that low expression of Ki67 was associated with the nuclear distribution of EBNA1 in the NPC biopsies (Fig. 9a). As shown in Fig. 9b, EBNA1 was observed to locate in the cell nucleus in two biopsies from total 21 patients with high Ki67 expression. However, among 22 patients with low Ki67 expression, EBNA1 was expressed in both nucleus and cytoplasm in 8 patients (correlation coefficient = 4.45, *p* = 0.037). These results suggested that EBNA1 was expressed in all EBV-positive NPC cancer cells and EBNA1 expression in nucleus probably indicates a low level of tumor growth progress.

## Discussion

As reported, EBV infection is significantly associated with increased risks and poor prognosis of NPC [36]. The main function of EBNA1 is to regulate DNA synthesis of EBV and maintain mitotic segregation of EBV episomes to daughter cells [2–4]. Our previous study has proposed EBNA1 as a new molecular target for antiviral and anticancer treating strategies [33–35, 37]. In this study, we found that a traditional extract of herbal medicine, triptolide, effectively suppresses NPC cell growth and induces NPC apoptosis in vivo and in vitro. Besides, the low-toxicity triptolide decreased EBNA1 expression in EBV-positive PBMCs from patients. Triptolide significantly decreased the expression of EBNA1 through a proteasome-ubiquitin pathway. Furthermore, we found that over-expression of EBNA1attenuates the caspase-9-dependent apoptosis induced by triptolide in NPC cells. In addition, EBNA1 was expressed in 100% NPC samples from patients and the nuclear distribution of EBNA1 indicated a low growth speed of NPC.

Triptolide is the most effective bioactive compound from *Tripterygium wilfordii* extracts [38]. Studies have suggested that triptolide killed cancer cells originated from blood, ovary, breast, lung prostate, and brain with IC_50_ values ranging from 2.5 to 50 nM [12]. Here, our results showed that the IC_50_ of triptolide acting on NPC cells were from 1.12to 75.56 nM. Our previous study found that the cell cycle of EBV-positive B lymphoma cells was retarded with a reduction in S phase [27]. Here, we observed that the cell cycles of EBV-positive epithelial NPC cells treated with triptolide were ceased in S phase, which indicates that triptolide may encumber DNA synthesis of EBV-positive NPC cells. Some studies have suggested that triptolide induces caspase-8, − 9, and − 3 activation and then activates downstream PARP in different cells [24, 25]. However, more evidence indicated that the essential apoptotic pathway is mitochondrial pathway rather than death receptor pathway [12, 24, 39, 40]. Our findings suggested that NPC cells were induced to apoptosis through a caspase-9 pathway with triptolide treatment. P53 was reported to be necessary for triptolide to induce apoptosis in some cancer cells [41, 42]. Our results demonstrated that triptolide actives p53-dependent apoptotic pathway in NPC cells, which was similar to other cancer cells treated with triptolide [41, 42].

Triptolide was reported to cause transcription inhibition by targeting the largest subunit of RNA polymerase II (RPB1)-XPB and then induce apoptosis or immune/inflammatory responses [26]. Our previous study found that the transcription level of LMP1 was inhibited by triptolide in B lymphoma cells [27]. Interestingly, we first found that triptolide increased the transcription levels of EBNA1 in NPC cells, but decreased protein level of EBNA1. Our previous study has certificated that triptolide inhibited LANA1, a critical latency antigen protein of KSHV, in KSHV-associated primary effusion lymphoma cells through proteasome-ubiquitin pathway [28]. Hence, we speculated whether triptolide has the similar effect on EBNA1. Although the exact mechanism of how triptolide inhibits EBNA1expression still remains unclear, our results that triptolide induced EBNA1instability and degradation through proteasome-ubiquitin pathway suggested triptolide decreased EBNA1 expression through the post-translation pathway. Previous studies found that Gly-Ala repeat sequence plays important roles in inhibiting or abrogating EBNA1 from degradation by interfering its interaction with the 26S proteasome [43, 44]. However, these conclusions were challenged when EBNA1 was artificially fused an Ub to its N terminus, it was efficiently and completely degraded which suggested that GAr can’t prevent Ub-EBNA1 from being degraded. In addition, EBNA1 and EBNA1ΔGAr had the similar half-life, which suggested that ΔGAr does not increased the stability of EBNA1 [45–47]. Interestingly, our results showed that high concentration and long-time treatment of MG-132 significantly increased EBNA1 expression, suggesting that EBNA1 degrades through proteasome-ubiquitin pathway, which is consistent with the finding of Chrysoula and his colleagues [46]. Similarly, the studies have reported that triptolide could induce degradations of various critical proteins from host or virus by promoting proteasome-ubiquitin pathway, including α-synuclein [48] and Tat [49].

Apoptotic protease activating factor-1(Apaf1) and caspase-9 dependent mitochondrial apoptosis is activated to response to intracellular stress factors and ultimately leads to DNA damage and cell death. Our results firstly reported that EBNA1 inhibited the triptolide-induced apoptosis through inhibiting the caspase-9-dependent mitochondria apoptosis. Notably, our results showed that this inhibitory effect didn’t rely on EBV genome and suggested that EBNA1 is an anti-apoptosis protein acting on mitochondria apoptosis pathway. V-val EBNA1 is the predominant subtype in NPC patients living in southeast China [31, 32]. Hence, we compared the anti-apoptosis effect of V-val with that of P-ala subtype of EBNA1. As speculated, V-val EBNA1 had the anti-apoptosis effect similar to P-ala EBNA1. Our previous studies have reported that EBNA1 could assist malignance cells to inhibit berberine [34] or 17-DMAG [33]-induced cancer cells death. Here, both P-ala and V-val EBNA1 attenuated the killing effects of triptolide on EBV-positive and EBV-negative NPC cell lines, suggesting that targeting on EBNA1 is a substantial strategy of treating EBV-positive NPC. It is a further reasonable interpretation how EBNA1 resists NPC cells death. However, the exact molecular mechanism how EBNA1 inhibits procaspase-9 split into active caspase-9 is still unclear. In addition, proteomic methods revealed that EBNA1 binds to a cellular ubiquitin-specific protease -USP7/HAUSP. Because USP7can stabilize p53 and Mdm2 by interacting with these proteins, EBNA1 can competitively bind to the binding pocket in the N-terminal domain of USP7 and ultimately cause p53 destabilization in EBV-positive cells [9, 11]. By inducing degradation of PML proteins, EBNA1 binds to USP7 and the host CK2 kinase and recruits these proteins to PML nuclear bodies and causes PML nuclear bodies disruption [9, 10, 50]. These mechanisms contribute to elucidating why EBV-positive cancer cells can resist DNA damage and apoptosis after treatment with DNA damaging agents, which may result in the development of NPC and gastric cancer.

The effect of triptolide on EBV-positive NPC cells was further confirmed by the animal experiments, since HONE1/Akata cell-induced xenograft tumors in BALB/c nude mice were significantly inhibited by triptolide. One group found that the positive rate of EBNA1 was about 92.5% in Indian population [51]. Here, the expression of EBNA1in NPC biopsy of Chinese population was first investigated. Our present data showed a higher sensitivity (100%) in EBV-positive tumor tissue of Chinese people, which suggested that detecting EBNA1 by immunohistochemistry assay may be a possible method to determine EBV infection in clinical application. Furthermore, our results showed that there were two statuses of EBNA1 expression. At all the biopsies, EBNA1 was expressed in cytoplasm in cancer cells. However, EBNA1 could be expressed in both cytoplasm and nucleus in some cases (23.36%). These results which were consistent with previous studies suggested EBNA1 may exert different functions in tumor cells based on its various distributions [51, 52]. Importantly, the result that EBNA1 expressed in cells nuclear revealed a low proliferation speed of cancer cells. Limited studies have focused on the function of EBNA1 in cytoplasm. As a transcriptional factor, EBNA1 could inhibit NF-κB pathway in carcinoma cell lines by decreasing the phosphorylation of IKKα/β, and negatively modulate oncogenesis [8]. Ki67 expression is upregulated by EBNA1 in LCL cell lines [53]. It’s consistent with our results that EBNA1 expressed in the nucleus was associated with a low tumor proliferation. Interestingly, the expression of EBNA1 in cytoplasm can only be found in cells of squamous cell carcinoma. In contrast, the expression of EBNA1 was found in nucleus of normal tissues. However, due to the restricted numbers of clinical samples, much more clinical samples deserve to be examined to confirm the phenomenon.

Our previous studies have suggested that low-toxicity 17-DMAG reduced the expression of EBNA1 in LCLs and inhibited the growth of tumor cells [33]. Here in this study, our results found that low-toxicity triptolide decreased EBNA1 expression in EBV-positive PBMCs. Although triptolide showed a super anti-tumor activity compared with adriamycin or aclacinomycin in clinical research, serious toxicities restricted the further clinical application of triptolide [12]. Overall, our results have indicated that triptolide inhibits EBNA1 expression at a low concentration, suppresses growth of NPC cells but has no significant toxic effect on PBMCs. These results suggested that a safe concentration of triptolide can be used in treatment of NPC in the future.

## Conclusion

**Fig. 10:**
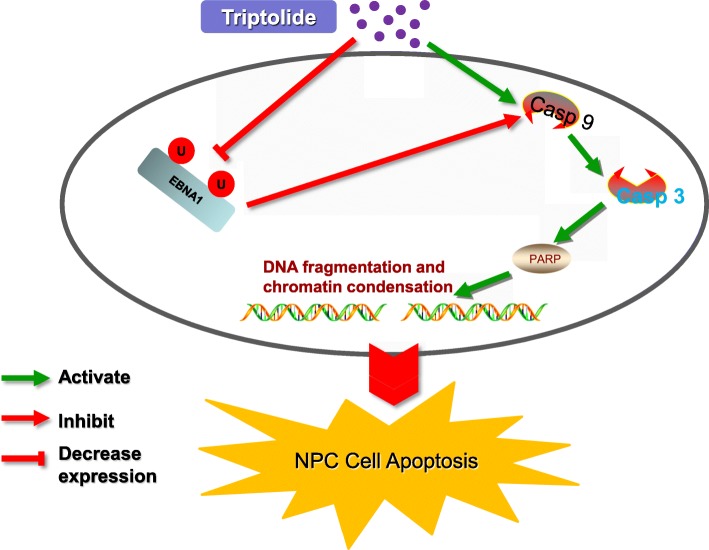
Schematic depicts a possible mechanism of triptolide inducing apoptosis in EBV-positive NPC cell. The caspase-9 activation plays a critical role in triptolide-induced mitochondria apoptosis in NPC cells. Triptolide inhibits EBNA1 expression through contributing to the proteasome-ubiquitin process and disturbing the stability of EBNA1, which promotes the activation of caspase-9, and induces the apoptotic effect of NPC cells

In summary, our data provide novel evidence that EBNA1 resists triptolide-induced NPC apoptosis through inhibiting the caspase-9 dependent apoptotic pathway, as depicted in Fig. 10. Triptolide decreases EBNA1 expression at a low-toxicity dose and inhibits EBV-positive NPC cells growth in vivo and in vitro.

## Abbreviations

AP-1: Activator protein 1
Apaf1: Apoptotic protease activating factor-1
ATF2: Activating transcription factor
CCK-8: Cell Counting Kit-8
CHX: Cycloheximide
CK2: Casein kinase 2
DMSO: Dimethylsulfoxide
EBER: EBV-encoded small RNA
EBNA1: Epstein-Barr nuclear antigen 1
EBV: Epstein-Barr virus
IKK: I-kappaB kinase
KSHV: Kaposi’s sarcoma-associated herpesvirus
LANA1: Latency-associated nuclear antigen 1
LMP: Latent membrane protein
NF-κB: Nuclear factor kappa B
NPC: Nasopharyngeal carcinoma
*Ori*P: Latent origin of replication
PBMC: Peripheral blood mononuclear cell
PML: Promyelocytic leukemia
RPB1: RNA polymerase II
SB: Sodium butyrate
SP1: Specificity protein 1
TP: Triptolide
TPA: 12-O-tetradecanoylphorbol-13-acetate
USP: Ubiquitin-specific protease
